# Influence of hydrological pathways on dissolved organic carbon fluxes in tropical streams

**DOI:** 10.1002/ece3.2543

**Published:** 2016-12-18

**Authors:** Eline Nayara Dantas da Costa, Jéssica Carneiro de Souza, Marilane Andrade Pereira, Marcelo Friederichs Landim de Souza, Weber Friederichs Landim de Souza, Daniela Mariano Lopes da Silva

**Affiliations:** ^1^ UESC/Laboratório de BiogeoquímicaUniversidade Estadual de Santa CruzIlhéusBABrazil; ^2^ INT/Divisão de Química AnalíticaLaboratório de Química Analítica InorgânicaInstituto Nacional de TecnologiaRio de JaneiroRJBrazil

**Keywords:** Atlantic forest, cacao, carbon, organic matter, watersheds

## Abstract

Water flow pathways and water balance are fundamental components for understanding the dynamics of C in the soil/water interface of small basins. The objective of this study was to describe the seasonal variations and estimate the annual balance of dissolved organic carbon (DOC) by comparing two tropical microbasins (preserved forest—PF and cacao plantation—CP). Twenty‐one weekly collections were conducted from September to December 2012 and from April to June 2013. The calculation of the partial balance considered precipitation (P) as inflow and the stream as outflow. The samples were filtered and analyzed using a TOC analyzer. Overall, the DOC was higher CP compared with FP. The behavior of both venues showed that rainy season caused an increase in concentrations in the overland flow (OF) and in the stream, and a decrease in the precipitation (P) and in the throughfall (T). In the CP, the outflow and the soil were chiefly responsible for the high DOC concentrations in the stream, when compared to the PF, which is the result of constant OM decomposition. Soil composition contributes to the control of DOC consumption in each type of soil. The balances were negative in both microbasins, although losses were higher in the AFS (agroforestry systems) when compared to the PF, especially during rainy seasons (−8.98 and −3.05 kg ha^−1^ year^−1^, CP and FP, respectively). Thus, the high annual loss of DOC in the CP of the microbasins during the rainy season indicates changes in ecosystem metabolism due to the vegetation cover and to the interactions with the soil.

## 1 Introduction

The monitoring of C cycling in regions covered by forests involving organic matter fluxes associated with hydrological pathways, soil, and vegetation helps to clarify the status of an area (Andrade et al., 2011; Markewitz, Davidson, & Moutinho, 2004; Schroth et al., 2001). In this case, regional balances of C and nutrients with inflow–outflow are useful indicators of the imbalances caused by changes in soil use, which are measured in spatial and temporal scales (Meyer & Tate, 1983). By measuring dissolved C export flows of inland waters, it is possible to evaluate the changes in the biogeochemical cycles caused by environmental alterations (Guo et al., 2007).

The organic carbon cycling is directly related to leaching process and decomposition of the organic matter (Creed, Webster, Braun, Bourbonnière, & Beall, 2013; Gergel, Turner, & Kratz, 1999). The dissolved organic carbon (DOC) comprises a fraction of water‐soluble organic matter (Michalzik & Matzner, 1999) that circulates in the hydrological pathways in labile and refractory forms from plant and animal decomposition, as well as the products for these excreted (David & Vance, 1991). The precipitation network and seasonal variations organic matter flows act as the main driving forces in the environmental C dynamics (Villela et al., 2012).

The organic carbon from the atmosphere is transported to the earth's surface through precipitation, which removes the dry material and wet biogenic and anthropogenic deposited in the atmosphere. This forms the major pathway for the removal of organic carbon from the atmosphere, affecting both atmospheric and landscape processes (Lavorivska, Boyer, & DeWalle, 2016). The redistribution of rainfall in the forest allows water to be enriched with nutrients being carried to ground by throughfall and stemflow, providing spatially different amounts to the forest floor (Germer et al., 2007; Germer et al., 2010; Levia & Frost, 2003). The pathways such as overland flow and soil solution represent a major flux of C and nutrients from terrestrial to aquatic systems (Raymond & Bauer, 2001; Wilson & Xenopoulos, 2009). Recent researches have been demonstrating the important links among DOM, ecosystem nutrient losses, and ecosystem C balances. At regional to global scales, organic carbon transported through streams and rivers represents a globally significant carbon flux that has recently received considerable attention (Butman & Raymond, 2011; Cole et al., 2007).

Among the tropical ecosystems, the Brazilian Atlantic Forest is considered one of the largest tropical forests in the world (Villela et al., 2012) where the native forest cover has been reduced to <10% of the area (Faria, Paciencia, Dixo, Laps, & Baumgarten, 2007). Northeast of Brazil, part of the Atlantic Forest, was used for cacao plantations (*Theobroma cacao*), transforming the country one of the main producers in the world according to the Brazilian Institute of Geography and Statistics**.** Most of this production (70%) is derived from the use of agroforestry systems (cacao plantation) with perennial shades (Lobão, Setenta, & Valle, 2004) which make the biota remain remarkably well conserved (Faria et al., 2007; Sambuichi et al., 2012).

In this type of cacao plantation, the understory of primary or secondary forests is replaced by cacao, which greatly benefits from the shade of the canopy of large trees (Sambuichi et al., 2012). This production system tends to result in a positive relationship with the environment as it stops deforestation and generates large quantities of organic matter, thus promoting a large inflow of carbon (C) in the soil (Costa et al., 2004; Kirby & Potvin, 2007). The cacao plantation, when compared to more conventional agricultural methods, may present great potential as a carbon sink (Mbow et al., 2014; Schroth et al., 2011, 2013), with its quantity of C comparable to natural forests (Gama‐Rodrigues et al., 2010). Thus, the literature considers that the agroforestry systems (cacao plantation) have to include discussions on reducing emissions from deforestation and forest degradation (REDD +) (Schroth et al., 2013). However, most studies are limited to analyzing the capture of C by the soil and generated by vegetation (Barreto et al., 2010; Gama‐Rodrigues et al., 2010; Nair, Kumar, & Nair, 2009; Rita et al., 2011; Schroth et al., 2013; Somarriba et al., 2013), and ignore the metabolic responses generated by the hydrological and connectivity pathways (water/soil interface).

Thus, our objective was to (1) describe the seasonal and spatial variations of the DOC and (2) calculate the balance using annual DOC flow projections in each microbasin. Our hypothesis is that dissolved carbon fluxes in the cacao plantation (CP) will be similar to those found in the preserved forest (PF) in most of the hydrological pathways, as it is expected that the cacao plantation does not generate substantial changes in forest system.

## 2 Study Area

**Figure 1:**
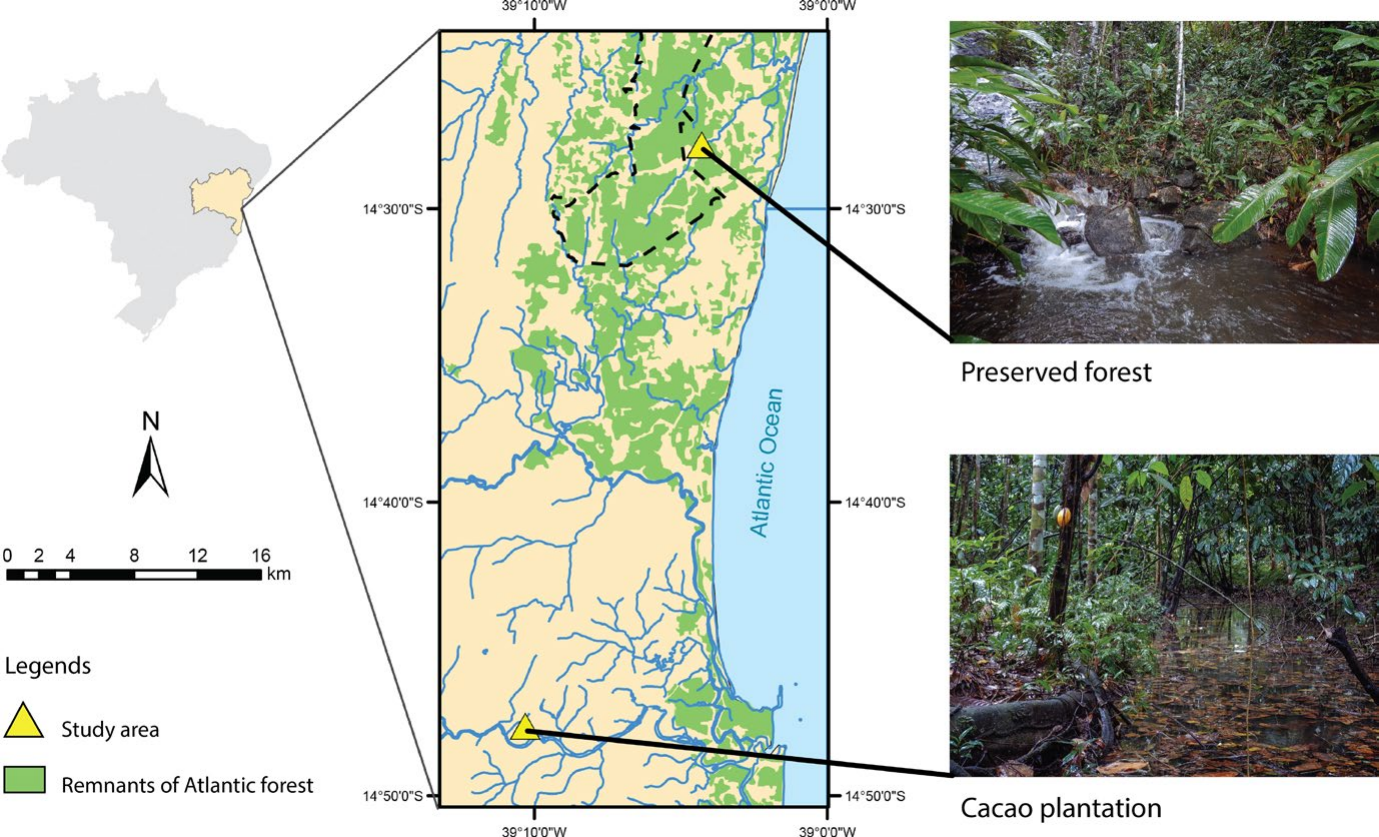
Location map of the microbasins of the preserved forest and of the cacao plantation

**Figure 2:**
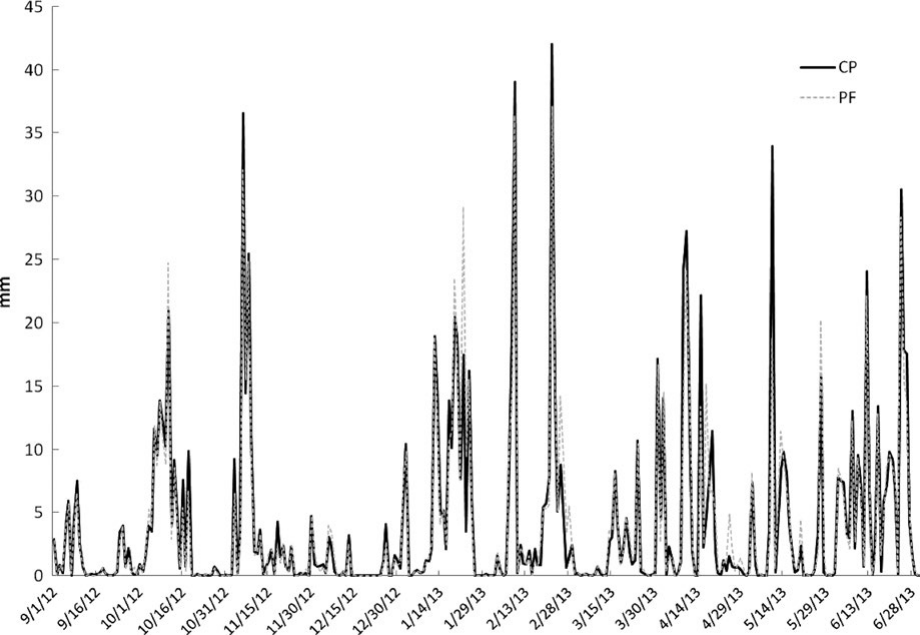
Daily rainfall in preserved forest (PF) and cacao plantation (CP) areas

The study was conducted in two microbasins with different soil uses (cacao agroforestry system and preserved forest), located at the coordinates S144738.2 W391019.5 and S8401012 W0492289, respectively, in the northeast of Brazil (Figure 1; Table 1). Annual rainfall from region is well distributed throughout the year, and relative humidity is above 80% (Asmar & Andrade, 1977; Sá et al., 1982). In precipitation, collectors were registered in PF 104.4 and 251.9 mm, in CP 103.2 and 246.8 mm to dry and rainy seasons, respectively (Table 1) and mean rainfall in the sampling period was 1,213 mm in CP and 1,188 mm in PF (Figure 2). The climate according to the Köppen classification is type AF (hot and humid without a well‐defined dry season).

**Table 1:** Characteristics of the watersheds—preserved forest (PF) and cacao plantation (CP)

ID	Lat/Long	Class/soil	Area (ha)	Land use	Precipitation (mm)	
					Dry	Rainy
PF	S8401012 W0492289	Oxisol	36.08	Preserved forest	104.4	251.9
CP	S144738.2 W391019.5	Dystrophic Ultisol	73.38	Cacao plantation	103.2	246.8

### 2.1 Preserved forest

The soil in the area is typically petroplinthic dystrophic latosol (Oxisol) (Silva, 2012) and a sandy texture (Moreau et al., 2006), with the proportion of 62% sand, 16% silt, and 22% clay and an acid pH around 4.5 (Souza, 2014). This area consists of a conglomeration of forests in various stages of regeneration (Martini et al., 2007). It is classified as a *Submontane Ombrophilous* Dense Forest that boasts a uniform canopy over 25 m tall with a few emerging individuals, many epiphytes, large vines, and a dense understory (Jardim, 2003).

### 2.2 Cacao plantation

The soil of the area is classified as typical Dystrophic Argisol to moderate eutrophic soil with medium clayey texture, with the proportion of 58% sand, 12% silt, and 30% clay. In the soil profile, the chemical characteristics show greater potential acidity (H + Al) in 40–60 and 60–80 cm and acid pH around 3.5 (Souza, 2014). The site is located on a slope of roughly 5%, and for 15 years, there has been no record of any acidity correction of the soil through typical CP procedures (introduction of inorganic carbon through the addition of gypsum and lime). Shading was inserted into a cacao monoculture, featuring 70% of cacao trees (*Theobroma cacau L*.) and 30% of jackfruit trees (*Artocarpus heterofolia*), cajá trees (*Spondias lutea*), coral trees (*Eritrina sp*.), trumpet trees (*Cecropia leucocoma*), and jequitibá trees (*Cariniana sp*.), among other species (Argôlo, 2009).

## 3 Materials and Methods

The hydrological pathways precipitation (P), throughfall (T), stemflow (SF), overland flow (OF), soil solution (SS at 10, 45 and 90 cm), and surface water (S) were studied using specific collectors in similar ways on the stream banks in the two microbasins. Sampling occurred weekly during two periods between 2012 and 2013, totaling 21 weeks. Rainfall events were monitored and the accumulated volume of each week was used to define the periods as dry and rainy (Table 1). These data were obtained from the website of the real‐time climate monitoring program for the northeast—Proclima. Abiotic parameters, including pH and electrical conductivity, were measured in the field using a portable WTW pH/Cond. 340 Sensor. The water samples were collected and preserved until they could be moved to the laboratory.

A collector was installed 1 m above the ground in an open area adjacent to the area with vegetation to measure the precipitation. The collector consisted of a PVC tube that covered an area of 0.14 m^2^. At one end of the tube, a funnel was attached and connected to a hose that led to 10‐L collectors, which are the same as those used for measuring throughfall. Five of these collectors were installed under the vegetation at distances of 5 m. The stemflow collectors were fixed to trees with DBH ≥ 15 cm of diameter on a plot of 10 × 10 m. These collectors consisted of a plastic hose glued around the trees with expanding polyurethane foam, 1 m above ground, linked to a 5‐L container. The overland collector was made of PVC with an area of 1.5 m and six were installed close to the soil in each basin. It consists of PVC tubes (150 cm in width and 60 mm) with side holes to collect and lead the water to a PVC tube that stores the water. The collector received protection to prevent the entry of branches, leaves, and small animals. The water samples were collected through a 60‐ml syringe. The stream outflow was collected manually through a 60‐ml syringe previously washed. In total, six individual tension lysimeters were installed. In each basin, three extractors were installed, one for each depth (15, 45, and 90 cm). The soil solution was extracted from the extractor using a 60‐ml syringe and hose. Manual pressure (vacuum) was applied with a syringe and needle prior to extraction. After lysimeters were installed and equilibrated for a 15‐day period, these first samples were discarded.

All samples from each collector were treated on site by filtration and 60 ml was filtered through a glass microfiber membrane (pore: 0.7 μm, precalcined at 450°C for 4 h), and transferred to precalcined glass bottles and preserved using mercuric chloride (Hg Cl_2_) until analysis. The DOC analysis occurred in an inorganic analysis laboratory at the National Institute of Technology (INTC) in Rio de Janeiro, Brazil. The concentrations were determined using a total organic carbon analyzer with an infrared detector (Shimadzu model TOC 500A).

The weekly cumulative volume of P, T, SF, and OF in L (Vs) was converted to mm considering 1 m^3^ = 0.001 L and the area (A) of the specific collector in m^2^ (Equation (1)).

(1) $$\text{mm}=\frac{\text{Vs}*0.001}{A}$$

The differences in weekly volume by concentration in the collectors of the P, T, and SF were standardized with average weighted by volume (AWV) (Equation (2)), where [ ] is the sample concentration in mg/L, Vs is the sample volume in mm, and Vt is the total volume of weekly via in mm.

(2) $$\text{WAV}=\frac{\left[\right]*\text{Vs}}{\text{Vt}}$$

To calculate the flow of COD (F) in kg ha^−1^ year^−1^ (in WAV to P, T, and SF; mg/L to OF), the sample volume (Vs) in mm was multiplied by the specific collector area (A) where ha and time (t) 1 year in s (Equation (3)):

(3) F=COD∗Vs∗A∗t

To calculate the river flow (Fr), the weekly outflow in L/s was estimated using the Schreiber equation based on Smith, Crossland, and Crossland (1999), that uses temperature, monthly rainfall, watershed area, and percentage of watershed (equations (4) and (5))

(4) $$Q=A*\left(\frac{\text{Af}}{r}\right)*\left(\frac{r}{2.74}*\mathrm{Di}*10^{6}\right)$$

where *A* = watershed area; Af = monthly runoff; Di = number of days in the *i*th month; *r* = precipitation.

The flux of dissolved inorganic nutrients was obtained by multiplying discharge and concentration values dividing them by the area of drainage basin (A) according to the equation:

(5) $$\text{Fr}=\frac{\text{discharge}\;\times \;[]}{A}$$

To calculate the partial balance of the dissolved carbon, DOC flows during precipitation (P) were used as an inflow in kg ha^−1^ year^−1^, minus the outflow in kg ha^−1^ year^−1^, here considered as part of the stream (S) (Equation (6)):

(6) BUDGET=P−S

The nonparametric Mann‐Whitney *U*‐test with a *p *<* *.05 was used to compare averages between dry and rainy seasons, and linear regression was used to test the interaction between pathways with STATISTIC 6.0

## 4 Results

To characterize water quality in the studied pathways, we measured abiotic parameters pH and EC (Table 2). Although significant deviations were registered, the pH of most of the pathways fell within the range of slightly acid and neutral, or between 6.2 and 6.8. However, the CP, and specifically the surface water, showed a more acidic pH that varied moderately during the sampling period. Regarding conductivity, the pathways showed a marked variation in dynamics, justified by the high standard deviation. In this case, even if the CP has a larger quantity of ions in the pathway, particularly in the stream, the values found between the areas showed the same order of magnitude.

**Table 2:** Abiotic variables in the hydrological pathways of the microbasin in the PF (preserved forest) and in the CP (cacao plantation). With P: precipitation, T: throughfall, SF: stemflow, and S: stream. Mean values ± standard deviation; Cond. = electrical conductivity

Pathway	pH	E. Cond. (μS/cm)
PF		
P	6.6 ± 0.4	40.9 ± 35.1
T	6.5 ± 0.4	56.8 ± 46.7
SF	6.8 ± 0.8	34.6 ± 12.1
S	6.3 ± 0.4	42.5 ± 5.1
CP		
P	6.7 ± 0.4	39.2 ± 16.7
T	6.6 ± 0.5	68 ± 30.9
SF	6.2 ± 1.2	57 ± 17.7
S	5.8 ± 0.1	102 ± 18.6

*n*: P = 18 e 13; TF = 88 e 74; SF = 21 e 45; S = 16 e 17 (FN e CP, respectively).

**Figure 3:**
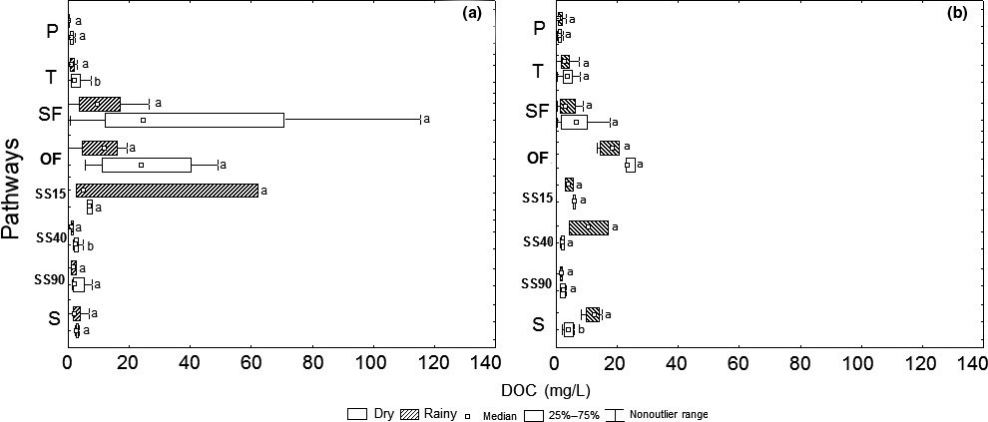
Dissolved organic carbon (DOC) distribution (mg/L) between dry periods and rainy periods in P: Precipitation, T: Throughfall, SF: Stemflow in volume‐weighted average—VWA, OF: Overland Flow, SS: Soil Solution (15, 45 and 90 cm), and S: Stream. a—Preserved Forest, b—cacao plantation. Different letters indicate different values between dry periods and rainy periods for the statistical Mann‐Whitney *U*‐test, with a *p* < .05

Comparing the two sites, the DOC concentration in P was similar between PF and CP in the dry season (1.2 and 1.3 mg/L, PF and CP, respectively); however, in the wet season, the DOC was two times higher in CP than FP (2.8 and 0.3 mg/L, CP and PF, respectively) (Figure 3). In CP was also observed higher DOC in T, OF, and S (*T* = 5.2 and 3.8 mg/L, OF = 27.8 and 22.1 mg/L, and S = 4.1 and 13.9 to dry and wet seasons, respectively) compared to PF (*T* = 3.7 and 1.7 mg/L, OF = 25.6 and 13.2 mg/L, and S = 2.6 and 3.1 to dry and wet seasons, respectively) (Figure 3). In PF, the SF presented the higher DOC concentration (41.4 and 17.4 mg/L, to dry and wet seasons, respectively) compared to PC (8.0 and 15.3 mg/L, to dry and wet seasons, respectively). Exception to CP stream (S), where DOC concentrations increased from 4.06 mg/L to 13.1 mg/L, most of the pathways did not show significant differences between the dry and wet seasons (*p* > .05). By observing the soil profile, it became clear that, in both areas, the average DOC concentrations decreased with the increase in depth (Figure 3a,b), with SS15: 19.6 and 5.9 mg/L, and SS90: 4.1 and 3.08 mg/L (for PF and CP, respectively). In both areas, the average concentration values were similar between SS45 and 90 cm (Figure 3), although comparing dry and rainy season, significant differences were found only in PF in SS45 (*p* < .05).

**Figure 4:**
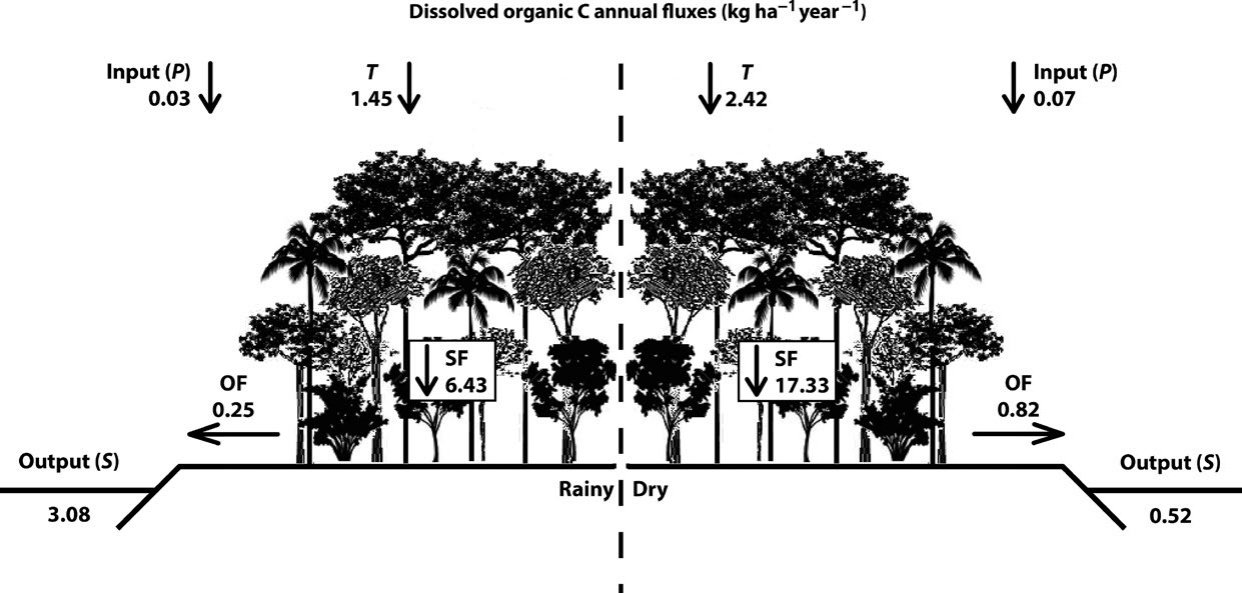
Annual estimates of DOC in kg ha^−1^ year^−1^ in dry and rainy seasons in P: precipitation, T: throughfall, SF: stemflow, OF: overland flow, and S: stream in the preserved forest—PF. The annual fluxes were calculated by equations (3) and (4) (see Section 3)

**Figure 5:**
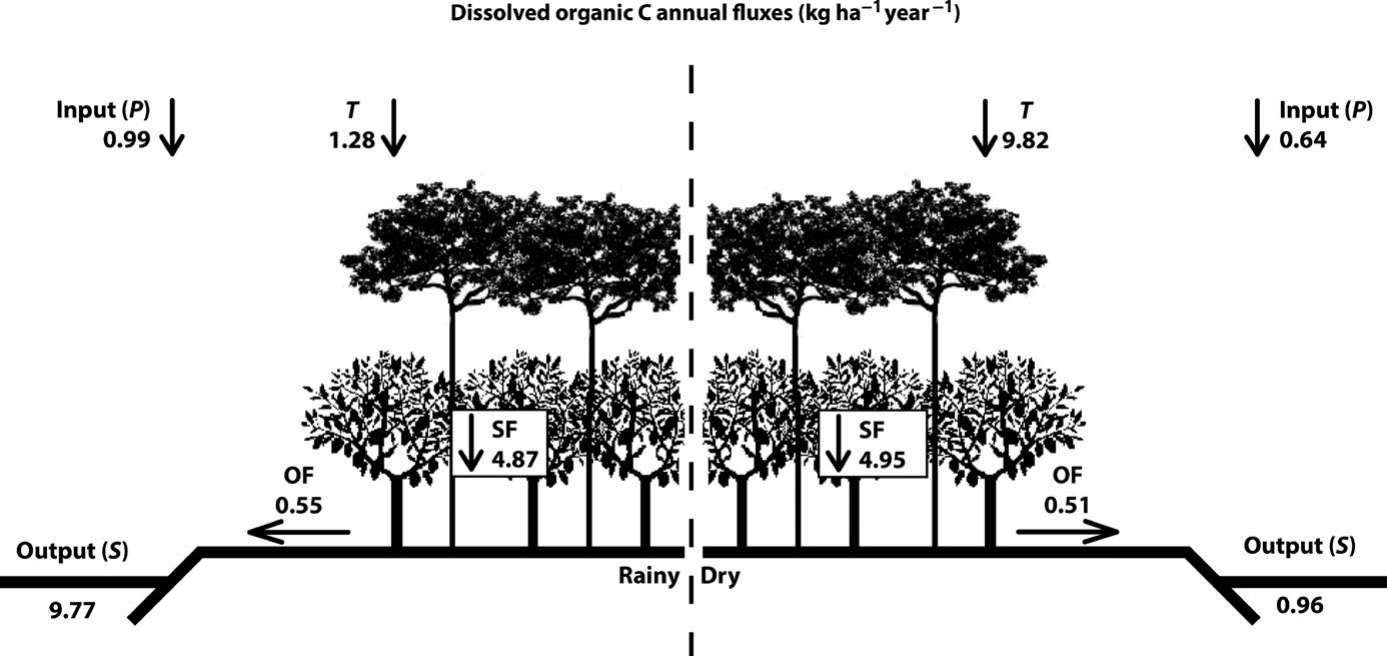
Annual estimates of DOC in kg ha^−1^ year^−1^ in dry and rainy seasons in P: precipitation, T: throughfall, SF: stemflow, OF: overland flow, and S: stream in the cacao plantation—CP. The annual fluxes were calculated by equations (3) and (4) (see Section 3)

The annual flows indicate that, between the dry and rainy periods, the DOC inflow through precipitation was lower in the PF (Figure 4) than in the CP (Figure 5). The values for the dry season were precipitation: 0.82 and 0.04 kg ha^−1^ year^−1^, and in the rainy season, the values were precipitation: 0.88 and 0.04 kg ha^−1^ year^−1^ (for PF and CP, respectively). Concentrations of DOC in throughfall (TF) were greater than those in precipitation (P) (Figure 5) where the concentrations exceeded the PF with a significant difference between the seasons (*p* > 0.05). The values in the dry period were throughfall: 2.42 and 2.77 kg ha^−1^ year^−1^, and in the rainy period, the values were throughfall: 1.45 and 1.28 kg ha^−1^ year^−1^ (for PF and CP, respectively). Of the pathways above ground, the stemflow was primarily responsible for transporting DOC to the soil (Figures 4 and 5). Stemflow represents the most significant route for DOC transportation to the ground, with 6.43 and 4.87 kg ha^−1^ year^−1^ (for PF and CP, respectively). In the dry season, the stemflow transported three times more in the PF when compared to the CP (stemflow: 17.73 and 4.95 kg ha^−1^ year^−1^ for PF and CP, respectively). During the rainy season, total DOC circulation in the pathways above ground was similar for both areas. Regarding the stemflow in PF, flows varied between periods, with larger flows occurring in the dry season, with 0.82 kg ha^−1^ year^−1^, and in the rainy period, with 0.25 kg ha^−1^ year^−1^ (Figure 4), while the CP transportation of DOC was about 0.5 kg ha^−1^ year^−1^, regardless of the season (Figure 5).

In the PF, both periods showed an inverse relationship between stream DOC and SS90 cm (*r* ^2^ = .54, *p* < .05); thus, the stream was directly related to the stemflow only in the rainy season (*r* ^2^ = .71, *p* < .05) (Table 3). The CP during the rainy season showed a DOC in the stream that was directly related to both SS90 cm (*r* ^2^ = .91, *p* < .05) and the stemflow (*r* ^2^ = .51, *p* < .05) (Table 3). In the comparison between the streams, an increased availability of DOC was observed in the CP and the flow was greater than in the PF, with outflows of 9.77 and 3.8 kg ha^−1^ year^−1^ in the rainy season and 0.77 and 0.52 kg ha^−1^ year^−1^ in the dry season for CP and PF, respectively. Using the partial balances, we observed that both areas showed negative balances during the periods (Figures 4 and 5). In the rainy season, the DOC loss was −3.05 and −8.89 kg ha^−1^ year^−1^, and in the dry season, the DOC loss was −0.45 and −0.13 kg ha^−1^ year^−1^ for the PF and CP, respectively.

**Table 3:** Stream DOC in relation to overland flow and SS90 during rainy and dry seasons

	PF		CP	
	OF	SS 90	OF	SS 90
Rainy				
S	*p* = .0066	*p* = .0113	*p* = .0288	*p* = .0010
	*r* ^2^ = .7995	*r* ^2^ = .6838	*r* ^2^ = .5179	*r* ^2^ = .9515
	*y* = −0.0122680362 + 0.129091841*X	*y* = 2.58659774 – 0.138790949*X	*y* = 3.3846719 + 0.23106043*X	*y* = −6.47686633 + 9.73205208*X
Dry				
S	*p* = .6337	*p* = .0229	*p* = .4267	*p* = .5954
	*r* ^2^ = .0279	*r* ^2^ = .5461	*r* ^2^ = .0923	*r* ^2^ = .0423
	*y* = 8.32246644 + 0.4799047*X	*y* = 15.3401714 – 3.7803487*X	*y* = 4.6294893 – 0.0692265953*X	*y* = 5.2253754 – 0.451090851*X

PF, preserved forest; CP, cacao plantation.

The *r* ^2^ and *p*‐value represent the simple linear regression between the variables.

## 5 Discussion

The DOC concentrations found in the pathways aboveground indicate that the availability of dissolved organic matter (DOM) generated by the litterfall represents an important source of DOC in both microbasins.The prevalence of DOC was found in a preserved area of the Atlantic Forest and the authors attributed these results to the natural conditions of the streams, which are mainly influenced by the vegetation of the canopy and temporal variations (Andrade et al., 2011). Soil organic matter (SOM) leaching is responsible for the control of numerous environmental processes, including the availability of DOC in aquatic ecosystems (Aitkenhead‐Peterson, McDowell, & Neff, 2002; Bolan et al., 2011), and acts as an energy source for the metabolism of these ecosystems (Amon & Benner, 1996; Cole et al., 2007; Jansen, Kalbitz, & McDowell, 2014) high proportion of DOC in relation to total C. In this study, the high proportion of DOC in relation to total C suggests that the decomposition process of fresh OM is constant , and this is followed by the release of labile OM (proteins) during the decomposition of the newly deposited litter. The benefits of the CP are based in the ability to stock C in the soil through the vegetation cover (Gama‐Rodrigues et al., 2010; Montagnini & Nair, 2004; Nair et al., 2009; Oelbermann & Voroney, 2007; Rita et al., 2011). However, the DOC proportion in the CP and the PF differed from the results found in environments where production causes a relatively greater environmental disturbance, such as the sugarcane plantations in the Brazilian Cerrado studied by Silva et al. (2007). During the dry season, the greater availability of refractory OM in the litter and the decrease in the volume of rainfall contributed to a decrease in DOC mainly in PF.

In this study, the suggests that the decomposition process of fresh OM is constant and certainly that this is followed. This was also observed by by Shibata et al. (2001) and Singh et al. (2014) in regions where the SOM was affected by the season in hydrological pathways in temperate forests. These authors reported that DOC concentrations were lower during winter due to decreases in the falling of leaves and branches. Unlike the findings of this study, in these works, the amount of more processed litter is lower than the amount of fresh litter due to the rapid consumption of OM by the microbial community and the predominant decomposition of a more refractory SOM that contributes to an increase in the DIC in relation to the DOC (Duan, Delaney‐Newcomb, & Kaushal, 2014; Singh et al., 2014).

The seasonality of precipitation in DOC concentration could reflect changing inputs from terrestrial vegetation and possibly differences in anthropogenic emissions during the course of a year (Willey, Kieber, Eyman, & Avery, 2000). The average concentrations in the precipitation of the studied areas correspond to the DOC patterns found in temperate and tropical forest climates (Lavorivska et al., 2016; Likens, Edgerton, & Galloway, 1983; McDowell, 1998; Möller, Kaiser, & Guggenberge, 2005). In these studies, the availability of organic and inorganic compounds deposited in the atmosphere decreases with the increases in intensity and frequency of rainfall, indicating that, during the winter when the frequency of rainfall increases, there is an increase in atmosphere washing and a decrease in DOC flow. However, our study showed that although the areas have similar rainfall volumes, in CP annual flows of P increased in the rainy season, while in FP decreased. Thus, it is believed that the existence of a highway near the sampling point of CP can justify this result. It is possible that the highway provides a continuum of aerosols from burning fossil fuels for transport atmospheric deposition.

As expected, the throughfall in both areas showed higher DOC concentrations than in precipitation, indicating the enrichment of DOC as water was intercepted by the vegetation. In this case, the precipitation becomes enriched with nutrients (Germer et al., 2007) due to the leaching of organic compounds derived primarily from plant tissues and the dissolution of soluble compounds deposited or produced by the surface of the leaves (Möller et al., 2005). In both areas, DOC concentrations were within the average recorded by Möller et al. (2005) in different landscapes of Thailand, although much lower than the average reported in subtropical forests by Liu and Sheu (2003) and in tropical rainforests (Jhonson et al., 2006; Markewitz et al., 2004; Tobón, Sevink, & Verstraten, 2004). This variation can be explained by the differences in the architecture and composition of the vegetation coverage, as well as by the different precipitation regimes identified among the landscapes, which are the main control factors of DOC concentration in the throughfall (Currie et al., 1996; Liu & Sheu, 2003; McDowell & Likens, 1988; Shen et al., 2011). Following the water balance presented by Pereira (2014) for the same locations and time periods, we can conclude that the ability to retain water in the tree canopy is probably the main reason for the different DOC concentrations observed between the PF and the CP. Although the water balance may indicate the efficiency of the areas in rainfall uptake, the CP was identified by the higher potential of water retention of the vegetation and by the greater loss by capture when compared to the PF. In this case, the shape and size of the leaves of cacao crops in the CP may have favored the capture of droplets of low kinetic energy that enhance evaporation (Moura et al., 2009), which would make the throughfall the most enriched in DOC when compared to the PF due to the accumulation of material on the leaf surface.

The annual DOC flows revealed that in both the CP and the PF, stemflow (SF) contributed to the largest share of DOC in the vegetation transport to the soil, with higher concentrations in the PF when compared to the CP. However, DOC concentrations in the CP and in the PF were lower than those recorded in primary and secondary subtropical forests and in coniferous forests (Liu & Sheu, 2003). The transport of DOC and its concentration are primarily governed by the morphology of the bark, the trunk of the trees, and the water retention period of the vegetation (Inagaki, Sakai, & Ohnuki, 1995; Liu & Sheu, 2003). As stemflow represents a fraction of the precipitation over the vegetation (Levia & Frost, 2003), the differences found could be attributed to variations in the types of vegetation, which largely alter stemflow volume and, consequently, the DOC concentrations (Liu & Sheu, 2003). In this study, despite showing similar fractions in both areas, the minimum volumes required for the formation of stemflow in the PF and in the CP suggest differences in water retention by the vegetation (Pereira, 2014). Thus, unlike the CP, the abundance of epiphytes and creepers and the predominance of trees with rough trunks observed in the PF favored greater DOC leaching from metabolites and atmospheric deposition. The presence of termites in some specimens explains the high standard deviation found in DOC concentrations of the stemflow in the PF. On the other hand, in the CP, the prevalence of *Theobroma cacao*, which is constantly shedding its leaves, facilitates the formation of clearings, while smooth trunks with no other bonded species increase the outflow and the watering of organic material (Pereira, 2014) that result in smaller concentrations of DOC.

The influence of overland flow in DOC transport to the streams can be observed from the directly proportional relationships during the rainy season in the PF and in the CP. Overland flow, together with soil drainage, is considered major route of DOC enrichment in streams, which represents the result of the production, absorption, and water transport in terrestrial components, especially the subsurface layer of the soil (Eckhardt & Moore, 1990; Jansen et al., 2014). While DOC flows of overland flow in the PF varied between the dry and rainy seasons, in the CP, contributions were similar, maybe resulting from the different water movement behavior between the soils of both areas. The movement of water in the soil is also related to soil texture, because unlike FP, the highest percentage of clay in PC (Table 2) can impede water infiltration resulting in similar surface flow between periods. The CP probably retains water in the first centimeters of the soil and the changes along the profile are reduced, thus decreasing the processes of DOC consumption, which, unlike the PF, is not influenced by rainfall volumes. On the other hand, DOC flows in overland flow found in the rainforest exhibit a strong seasonal pattern (Jhonson et al., 2006). This reflects the SOM content transported from the infiltration to the outflow into the stream throughout, which is dependent on the dynamics of the litter and the biomass above ground. In a comparison between cacao and other tropical crops, *Theobroma cacau* showed a concentration of roots with a diameter <3 mm at a depth of 0–10 cm, which affects the presence of pores with decreasing hydraulic conductivity and can contribute to the increase in DOC in this soil layer (Augusto, Martins, & Góes, 2004; Martins & Augusto, 2012). According to Jansen et al. (2014), hydrological regimes often control the OM transport in the soil and its interactions possibly affect the soil hydrology.

The reduction of DOC concentration from SS15 to SS90 observed in both areas indicates that OM processing is more effective from the surface to the deeper soil layers. They highlighted that microbial activities are more intense in the organic horizon than in the mineral soil horizon. This occurs because the first layers depend on DOC availability caused by decomposition of the litter. This process decreases as the profile progresses due mainly to the DOC sorption that increases with depth (McDowell and Likens, 1998) and the microbial functional diversity, which changes according to the quality of OM that, in turn, gets more degraded with increased depth. A similar dynamic was found by Fujii, Uemura et al. (2009), Fujii et al. (2011), Kalbitz et al. (2000), Möller et al., 2005, Singh et al. (2014), Toosi, Schmidt, and Castellano (2014) in temperate and tropical forests.

The soils of the areas were considered acidic, with a pH of around 4 at 0–20 cm; however, in the PF, while the pH increases in the soil profile, CP decreases. According to Do Nascimento et al. (2008), Fujii, Funakawa et al. (2009), Fujii et al. (2011), wet tropical regions present the highest concentrations of DOC in the organic layer due to an acidic pH soil, which is generally below 4.3. These authors reported that in the organic horizon, DOC availability is connected to the composition of the leaf and the large accumulation of humus in the first centimeters of soil, which, associated with the acidic pH, can stimulate the production of recalcitrant DOC. Below the organic layer, DOC concentrations decrease due to the control especially the adsorption through clay materials and adsorbent hydroxides (Al and Fe) (Kaiser & Zech, 2000; Kalbitz et al., 2000; Qualls & Haines, 1992). The decrease of DOC in SS90 could be associated to differences observed between the areas in regards to the potential acidity (H + Al) and Cation exchange Capacity (CEC). Souza (2014) studying soil characteristics in the same areas reported lower values of CEC and potential acidity (H + Al) in the soil of the PF.

Besides, the predominance of DOC over the total C available in the SS90 of the PF may also indicate that the distribution of roots along the soil profile is more uniform than in the CP. In the soil profile, DOC flows from the organic horizon, root debris, and the source material (clay and sand) control the presence of DOC, which varies according to the different compositions and depths (McDowell, 1998; Neff & Asner, 2001; Tobón et al., 2004). The small difference between the sand and clay percentages allowed for the composition of the soils to be classified as medium sandy texture and medium clay for the PF and the CP, respectively (Souza, 2014).

The inverse relationship between the SS90 cm and the DOC in the stream during both periods in PF may be explained by this difference found, because in this area, the water presented greater infiltration potential in comparison with the CP. This indicates higher water movement in the soil profile of the PF, so DOC is transported to greater depths and consumed before reaching the stream, as found by Shibata et al. (2001) in the temperate forest, where the high hydraulic conductivity is attributed to the sand content. According to Augusto et al. (2004) and Martins and Augusto (2012), in sandy soils, macropores promote better drainage, enabling thicker and deep roots, while in clay soils, the predominance of micropores allows for thin and shallow roots that reduce hydraulic conductivity. Therefore, we suggest that higher DOC concentrations in the CP at SS90, when compared to the PF, are also influenced by the presence of sand, which has a low potential for adsorption, and are influenced by the deep DOC sources derived from OM transport in the soil matrix or the roots. A similar pattern was found by Fujii et al. (2011), when comparing tropical basins (sandy Oxisol soil and Argisol soil rich in clay) that showed a direct relationship between DOC availability and the presence of sand in the mineral horizon. This suggests that the constant deposition of litter, characteristic of *Theobroma cacau*, alongside the attributes of the top surface layer of the soil, affects the hydrology of the CP and contributes to greater DOC availability in the overland flow and at SS15 when compared to the PF.

During the rainy season, DOC transport by the streams was five and ten times higher (PF and CP, respectively) in comparison with the dry season in both areas; however, the annual flows in the PF accounted for a third of what was recorded in the CP. In contrast, DOC flows in the dry season were the same in the microbasins. The release of DOC in the streams depends on the production rate, the decomposition of the litter, and the SOM, which tend to decrease with the end of the rainy season (Jhonson et al., 2006), but also by leaching and absorption as the water flows through the organic horizon of the soil, surface, and subsurface outflow (Eckhardt & Moore, 1990). In both areas, the DOC flows were lower when compared to the preserved streams in the Atlantic Forest (Andrade et al., 2011) and the Amazon rainforest (Jhonson et al., 2006). These DOC dynamics were determined by the influence of certain factors such as climate, atmospheric deposition, geology, soil type, and vegetation coverage. However, DOC flows in the CP and in the PF are within the range of values of streams with different soil uses in Thailand (Möller et al., 2005) and Australia (Bass et al., 2014). In these studies, the mobilization of the C stock in the soil during the rainy season topped the effects of dilution expected by the increase in water volume, thus increasing DOC transport in the streams as the forests suffered modification by the type of land use.

In general, regional balance (considering only inflow and outflow) is regulated by soil properties (type, slope, and hydrology), vegetation coverage, and history of land use of the basin (Eckhardt & Moore, 1990; McDowell and Asbury, 1994). In this study, changes in vegetation and soil characteristics represented the main controlling factors of DOC losses each year by the microbasins. Regardless of the sampling period in the two areas, negative balances were found, with DOC outflow exceeding the inflow, which indicates that both the PF and the CP behaved as nonconservative systems. The disturbance of forest ecosystems may initially result in the increased outflow of elements, showing that the recycling of nutrients is not being effective (Hobbie & Likens, 1973). Positive balances reported by Möller et al. (2005) in natural tropical, regenerating and plantation forests in Thailand, indicate that DOC introduced by precipitation is incorporated by the systems, thus ensuring efficient C mineralization and ecosystem balance.

In spite of the negative balances in the areas during the rainy season, the loss of DOC that was three times higher in the CP than in the PF can be explained by the direct relationships found between the overland flow and the SS90 with the stream. As discussed above, these pathways highlight the important role the vegetation cover and the soil potential have as DOC loss controllers. In the CP, this was observed in the discrepancy between DOC transport by overland flow and by the stream, which emphasizes the soil as a main factor in the loss of DOC. Although the inverse relationship between the stream and the SS90 shows that, in the PF, much of the DOC is consumed in the soil before reaching the stream, thus reducing losses, the soil is also a major factor for the loss of DOC, as observed by the sharp rise in overland flow to the stream. In the dry season, contrary to the CP, the PF showed a decrease in DOC transport by the overland flow, suggesting that the greatest losses of DOC revealed in the balance are derived mainly from autochthonous material that supplies the DOC transport in the stream during this period.

## 6 Conclusion

The seasonal patterns of the PF and the CP were similar in the pathways above ground, with DOC values that decreased during the dry period in precipitation and throughfall and increased in overland flow and stream. In the soil, the DOC concentrations decreased with each increase in depth (SS15–SS90 cm) in both areas, but the DOC availability in the streams showed different results in the two areas, linked to soil properties and the presence of the cacao plantation (CP). The annual DOC flows indicate that during the dry season, the outflows are similar in both areas. In the rainy season, however, the presence of the CP influenced the loss of nearly three times more DOC than in the PF.

## Conflict of Interest

None declared.
